# Recent Advances in Visible-Light-Driven Photoelectrochemical Water Splitting: Catalyst Nanostructures and Reaction Systems

**DOI:** 10.1007/s40820-015-0063-3

**Published:** 2015-10-28

**Authors:** Xiaoping Chen, Zhixiang Zhang, Lina Chi, Aathira Krishnadas Nair, Wenfeng Shangguan, Zheng Jiang

**Affiliations:** 1 Research Center for Combustion and Environment Technology, Shanghai Jiao Tong University, Shanghai, 200240 People’s Republic of China; 2 Faculty of Engineering and the Environment, University of Southampton, Highfield, Southampton, SO17 1BJ UK; 3 School of Environmental Science and Technology, Shanghai Jiao Tong University, Shanghai, 200240 People’s Republic of China

**Keywords:** Photoelectrochemical water splitting, Nanostructures, Reaction system, Heterojuction, Hybrid systems

## Abstract

Photoelectrochemical (PEC) water splitting using solar energy has attracted great attention for generation of renewable hydrogen with less carbon footprint, while there are enormous challenges that still remain for improving solar energy water splitting efficiency, due to limited light harvesting, energy loss associated to fast recombination of photogenerated charge carriers, as well as electrode degradation. This overview focuses on the recent development about catalyst nanomaterials and nanostructures in different PEC water splitting systems. As photoanode, Au nanoparticle-decorated TiO_2_ nanowire electrodes exhibited enhanced photoactivity in both the UV and the visible regions due to surface plasmon resonance of Au and showed the largest photocurrent generation of up to 710 nm. Pt/CdS/CGSe electrodes were developed as photocathode. With the role of p–n heterojunction, the photoelectrode showed high stability and evolved hydrogen continuously for more than 10 days. Further, in the Z-scheme system (Bi_2_S_3_/TNA as photoanode and Pt/SiPVC as photocathode at the same time), a self-bias (open-circuit voltage *V*_oc_ = 0.766 V) was formed between two photoelectrodes, which could facilitate photogenerated charge transfers and enhance the photoelectrochemical performance, and which might provide new hints for PEC water splitting. Meanwhile, the existing problems and prospective solutions have also been reviewed.

## Introduction

The energy consumption nowadays to maintain modern lifestyle of mankind mainly relies on primary fossil fuels such as oil, coal, natural gas, etc. However, the fossil fuels are suffering from accelerated depletion and bringing about serious environmental issues and threats to global climate. It is a mission of international scale to explore and utilize alternative energy to compensate for the consumption of fossil fuels and mitigate the corresponding climate changes. Among various alternative energies, hydrogen has been considered as a promising candidate to solve aforementioned problems because it is a source of green and renewable energy. There are a variety of strategies for hydrogen production, such as electrolysis, thermal water splitting, cracking of petroleum, hydrocarbon reforming, etc. However, these techniques are either costly or rely on fossil fuels. In 1972, Fukushima and Honda discovered photoelectrochemical (PEC) water splitting in which hydrogen and oxygen were released, respectively, from titanium dioxide (TiO_2_) photoelectrode and Platinum (Pt) counter electrode under ultraviolet (UV) light irradiation [1], revealing the potential of solar energy water splitting to produce sustainable hydrogen. This discovery stimulated great interest to explore effective photoelectrode materials for solar energy hydrogen generation via solar energy water splitting which is not only a clean process but also stores solar energy in hydrogen [2–4]. However, the widespread application of PEC water splitting still demands great efforts in the discovery of effective photocatalysts and coating process.

In comparison with photocatalytic water splitting using heterogeneous powder semiconductors, PEC water splitting possesses great advantages in (i) the external or self-bias voltage can suppress recombination of photogenerated charge carriers and thus improve the separation and transfer of excited electron–hole pairs of the photocatalysts; (ii) hydrogen and oxygen can be easily separated via collection at different photoelectrodes; (iii) semiconductor films are coated on the conductive substrates, which favors scale up for industrial application in the future; and (iv) last, but not the least, it does not need stirring, so it consumes less power relative to powder photocatalytic water splitting systems [2, 5]. In practice, the performance of PEC water splitting system is dominated by the properties of the semiconductor photocatalysts that harvest solar energy for hydrogen generation.

Various effective UV-light-responsive photocatalysts have been well established [6, 7], although most of them suffer from photocorrosion and are not active under visible light which accounts for 45 % energy of solar spectrum, nearly an order of solar energy in UV region. In the recent decades, designing visible-light-driven photocatalysts for water splitting represents a major mission for photocatalytic water splitting to maximize the solar energy conversion and storage. For instance, the metal sulfides have been found as a class of efficient photocatalysts but require sacrificial agents to reduce photocorrosion [8–12]. (Oxy) nitride semiconductors recently emerged as new type of phototcatalysts for visible-light-responsive photocatalytic water splitting [13–15], whereas they can respond to short-wavelength visible light with rather low solar energy conversion efficiency. Fortunately, the hybrid photocatalyst systems have demonstrated enhanced water splitting efficiency, which, however, require dedicated design and alignment of the corresponding photocatalytic materials [6].

In this study, we briefly overviewed the recent research advances in the field of hydrogen evolution from PEC water splitting, addressing the different PEC water splitting systems and corresponding electrode materials. Key issues and challenges involved in PEC water splitting systems and potential solutions were highlighted via the comparison of various photoelectrode materials and nanostructures.

## Working Principle of Photoelectrochemical Water Splitting

**Fig. 1:**
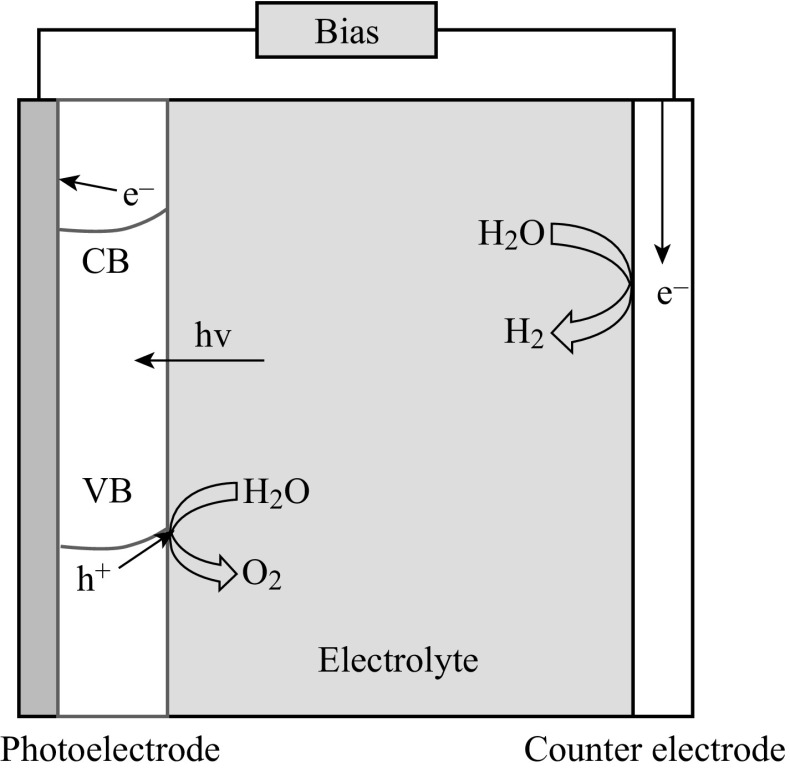
The schematic setup of PEC water splitting system

Loaded with semiconductor photocatalysts, the conductive electrode substrates can be metal plates, silicon substrates, or glass coated with conductive layers, such as fluorine-doped tin oxide (FTO) and indium tin oxide (ITO) [16–19]. In PEC water splitting system, the free energy change (Δ*G*) is 237.2 kJ mol^−1^ for converting one molecule of H_2_O–H_2_ and 1/2 O_2_ under standard condition. When a photoelectrode is immersed in an electrolyte solution, electron transfer takes place between the semiconductor and the electrolyte solution so that the Fermi level is equilibrated with the redox potential of electrolyte solution [15]. With n-type semiconductors as working photoanodes, for example, as shown in Fig. 1, photoexcited holes on the semiconductors would oxidize water and produce oxygen, while electrons are transferred to the counter electrode to generate hydrogen. Sometimes, an external voltage is needed to compensate for the potential deficiency, which can also accelerate the separation of excited charges. The electrolytes are essential in the PEC system for charge transfers, which usually are NaOH, Na_2_SO_4_, etc. [20, 21]. Some sulfides and organic agents can also serve as electrolytes [19, 22]; where there is no oxygen evolution, as water oxidation potential is more positive than the oxidation potential of these materials.

## Semiconductors Systems for PEC Water Splitting

Different semiconductor systems for PEC water splitting and their PEC performance are shown as in Table 1. They can be classified into three groups: (1) photoanode, (2) photocathode, and (3) Z-scheme system for PEC water splitting.

**Table 1:** Different photoelectrodes and their photoelectrochemical performance

Photoelectrode	Photocurrent (mA cm^−2^)	Experimental condition	Reference
TiO_2−*x*_C_*x*_ nanotube array	1.0	0 V versus Ag/AgCl, 1 M KOH(aq), 2500 W Xe lamp (100 mw cm^−2^), >420 nm	35
N-doped TiO_2_ nanotube arrays	0.182	0 V versus calomel electrode, 0.01 M Na_2_SO_4_ (aq), 250 W halogen lamp, >400 nm	36
S-doped TiO_2_ nanotube arrays	0.41	0.1 V versus SCE, 0.1 M Na_2_SO_4_ (aq), 50 W fiber optic illuninator, >400 nm	37
Au nanoparticle-decorated TiO_2_ nanowire electrodes	1.49	0 V versus Ag/AgCl, 1 M NaOH(aq), white-light illumination (100 mW cm^−2^)	40
Au decorated ZnO nanowire arrays	1.5	1 V versus RHE, 0.5 M Na_2_SO_4_ (aq), 300 W Xe lamp (100 mW cm^−2^), >420 nm	42
CdTe/TiO_2_	0.44	0 V versus Ag/AgCl, 0.6 M Na_2_S (aq), 300 W Xe arc lamp (6.0 W cm^−2^ >400 nm	43
CdS/TiO_2_	5.6	0 V versus Ag/AgCl, 0.1 M Na_2_S (aq), 300 W Oriel solar simulator (100 mW cm^−2^)	46
Bi_2_WO_6_/TiO_2_	0.014	1 V versus Ag/AgCl, 0.5 M Na_2_SO_4_ (aq), 300 W Xe lamp, >420 nm	47
CdS/TiO_2_/WO_3_	1.6	0 V versus Ag/AgCl, 0.05 M Na_2_S (aq), 300 W Xe lamp, >495 nm	50
Hydrogen-treated TiO_2_ nanowire arrays	1.97	−0.6 V versus Ag/AgCl, 1 M NaOH(aq), 150 W Xe lamp (100 mW cm^−2^)	52
N-doped ZnO nanowire arrays	~0.15	0.5 V versus NHE, 0.5 M NaClO_4_ (aq), white light source (100 mW cm^−2^)	56
WO_3_/BiVO_4_	0.8	0.5 V versus NHE, 0.5 M Na_2_SO_4_ (aq), chopped white light (100 mW cm^−2^)	58
FeOOH	10	0.55 V versus RHE, 1 M Na_2_CO_3_ (aq), 150 W Xe arc lamp (100 mW cm^−2^), >400 nm	60
IrO_2_·nH_2_O/TaON	~3.75	0.6 V versus Ag/AgCl,, 0.1 M Na_2_SO_4_ (aq), chopped visible light	65
IrO_2_-loaded Ta_3_N_5_	3.6	0.6 V versus Ag/AgCl, 0.1 M Na_2_SO_4_ (aq), 300 W Xe lamp, >400 nm	67
TiO_2_ nanoarrays sensitized with CdS quantum dots	3.98	0 V versus Ag/AgCl, 1.0 M KOH (aq), 300 W Xe lamp (100 mW cm^−2^), >420 nm	68
Pt–In_2_S_3_/CuInS_2_	−17.5 to −7.0	0 V versus RHE, 0.1 M Na_2_SO_4_ (aq), 300 W Xe lamp	74
Pt–CdS/CuGaSe_2_	−3.2	0 V versus RHE, 0.05 M Na_2_HPO_4_(aq) + 0.05 M NaH_2_PO_4_(aq), 150 W Xe lamp	18
p–n Cu_2_O homojuction	−0.2	0 V versus NHE, 0.5 M Na_2_SO_4_ (aq), 500 W Xe lamp	79
Pt/ZnO, Al_2_O_3_, TiO_2_/Cu_2_O	−7.6	0 V versus RHE, 1 M Na_2_SO_4_ (aq), 500 W Xe lamp (100 mW cm^−2^), visible light	80
Photoanode: TiO_2_	0.2	0 V versus Ag/AgCl, 1 M NaOH(aq), 500 W Xe lamp	83
Photocathode: CaFe_2_O_4_			
Photoanode: WO_3_	0.02	3 M H_2_SO_4_(aq), 250 W Oriel tungsten–halogen quartz lamp (200 mW cm^−2^)	84
Photocathode: GaInP_2_			
Photoanode: Bi_2_S_3_/TNA	1.6	0 V versus Ag/AgCl, 0.25 M Na_2_S + 0.125 M Na_2_SO_3_, Xe lamp (100 mW cm^−2^), >400 nm	19
Photocathode: Pt/SiPVC			

### Photoanode and Anodic Semiconductors

**Fig. 2:**
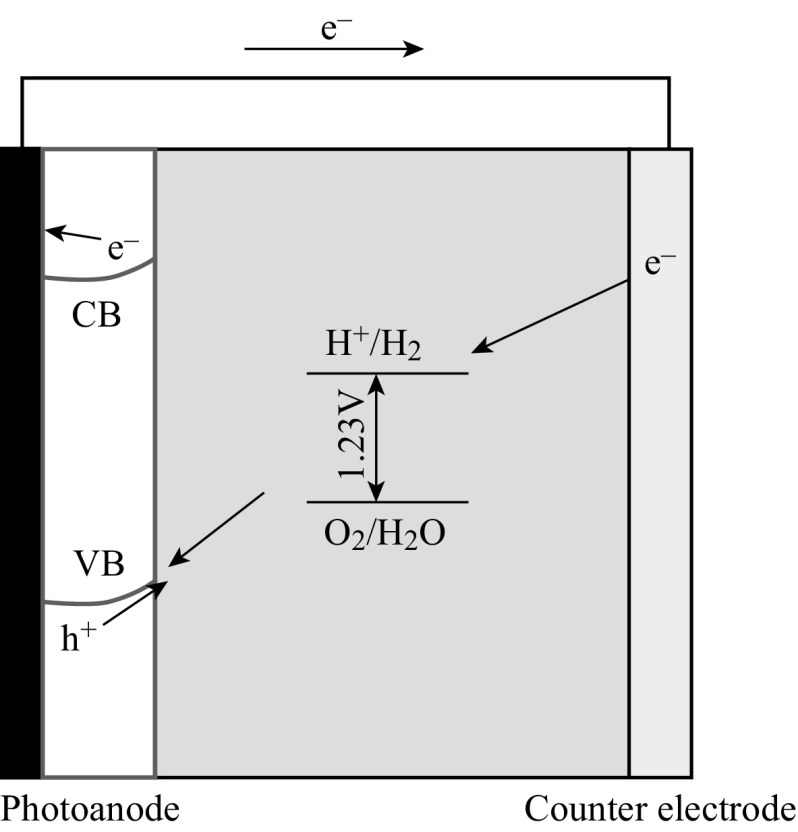
Semiconductors coated on substrate as photoanode for PEC water splitting [15]

**Fig. 3:**
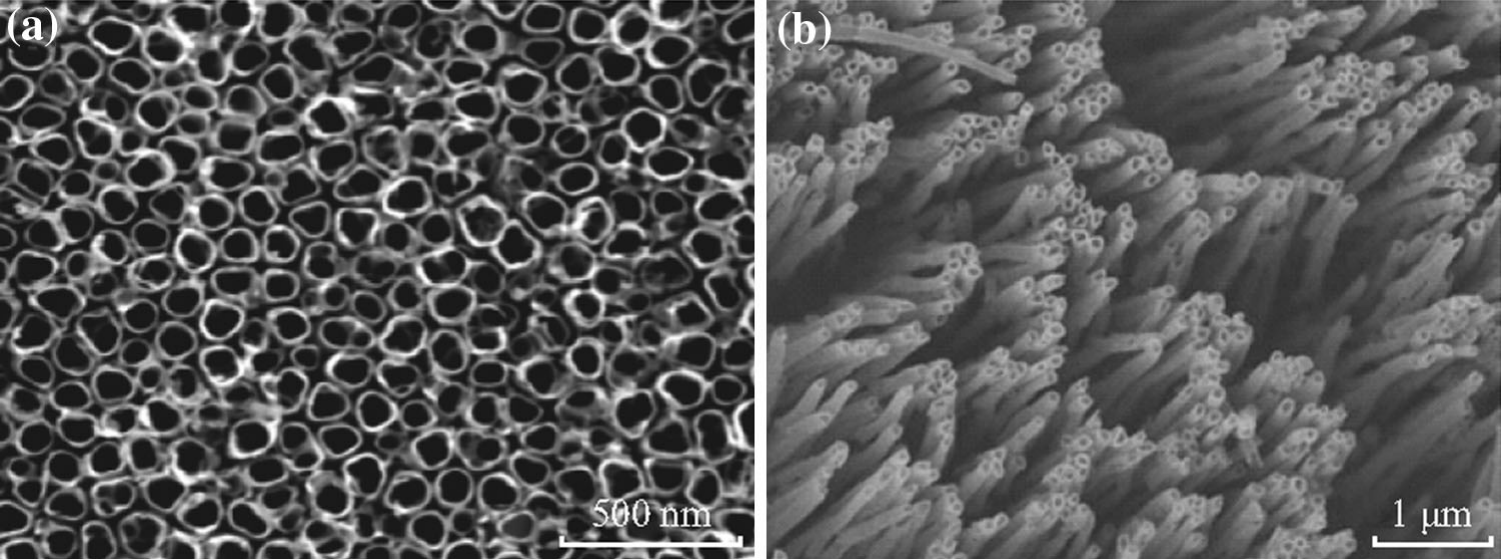
SEM images of titanium dioxide arrays [29, 34]

In the PEC water splitting setup, photoanode usually comprises n-type semiconductors coated on conductive substrates [15, 19]. Under light illumination, photoexcited holes accumulate on the surface of the photoanode semiconductors and are consumed in oxidation reactions, while electrons are transferred to a counter electrode via an external circuit as shown in Fig. 2 [15]. From the electrochemical potential point of view, the valence band edge of the photocatalysts should be more positive than the oxygen evolution potential enabling the photoanode to generate oxygen. As one of the significant advantages of PEC, external voltage bias may be applied to compensate for the potential deficiency and accelerate the separation of excited charge carriers, although zero bias is desirable once the PEC systems become well aligned with suitable semiconductor materials. Starting with traditional TiO_2_ photoanode, this section highlights the recent advances in photoanodes composed of one-dimensional (1D) TiO_2_ and various hybrid photoanode systems (Fig. 3).

Titanium dioxide (TiO_2_) is the most attempted n-type semiconductor for PEC water splitting due to its low cost, and better chemical and optical stability [23–26]. Among various nanostructured TiO_2_, 1D titanium dioxide nanotube arrays (TNAs) have gained much attention due to their intrinsic large surface area and unidirectional flow of charges [24, 27]. TNA can be easily fabricated via anodization of metal titanium foil or plate with great potential for large-scale application [24, 27–29]. 1D TiO_2_ nanowire arrays were also coated on transparent conducting oxide (TCO)-coated glass through a mild hydrothermal reaction [30]. The photoconversion efficiencies of N719 dye-sensitized solar cells fabricated with the 1D nanowire arrays can be up to 5.02 %, which is much higher than those fabricated with the simple TiO_2_ powders. Titanium dioxide itself shows low photoelectrochemical activity since it is not responsive to visible light due to wide bandgap (~3.2 eV). In order to enhance the visible light absorption, researchers have developed various strategies including doping TiO_2_ with metal or non-metal, construction of heterojunction, and hydrogenation, or creation of structural vacancies [31–33]. Doping with metal or/and non-metal (C, N, S, B, etc.) was started in the early-twenty-first century as an effective solution to narrow the bandgap of TiO_2_ for enhancing PEC efficiency [6, 34–39]. Metal doping and non-metal doping can lead to the conduct band and the valence band increases of acceptor, respectively, which narrowed the bandgap of the photocatalysts and make the photocatalysts respond to visible light. Park et al. prepared carbon-doped TiO_2_ nanotube arrays with high aspect ratios. The total photocurrent was more than 20 times higher than that with a P-25 nanoparticulate film under white-light illumination [35]. Recently, surface plasmon resonance has been applied in PEC water splitting with extend light absorbance in the entire UV–Visible region [40–42]. Surface plasmon resonance is an intrinsic property of metal nanoparticles, in which the oscillation frequency is highly sensitive to the metal size and shape as well as the dielectric constant of the surrounding environment. For instance, Au nanoparticle-decorated TiO_2_ nanowire electrodes showed the largest photocurrent generation at 710 nm and enhanced photoactivity across the entire UV–Visible region, which is due to the excitation of surface plasmon resonance of Au [40].

**Fig. 4:**
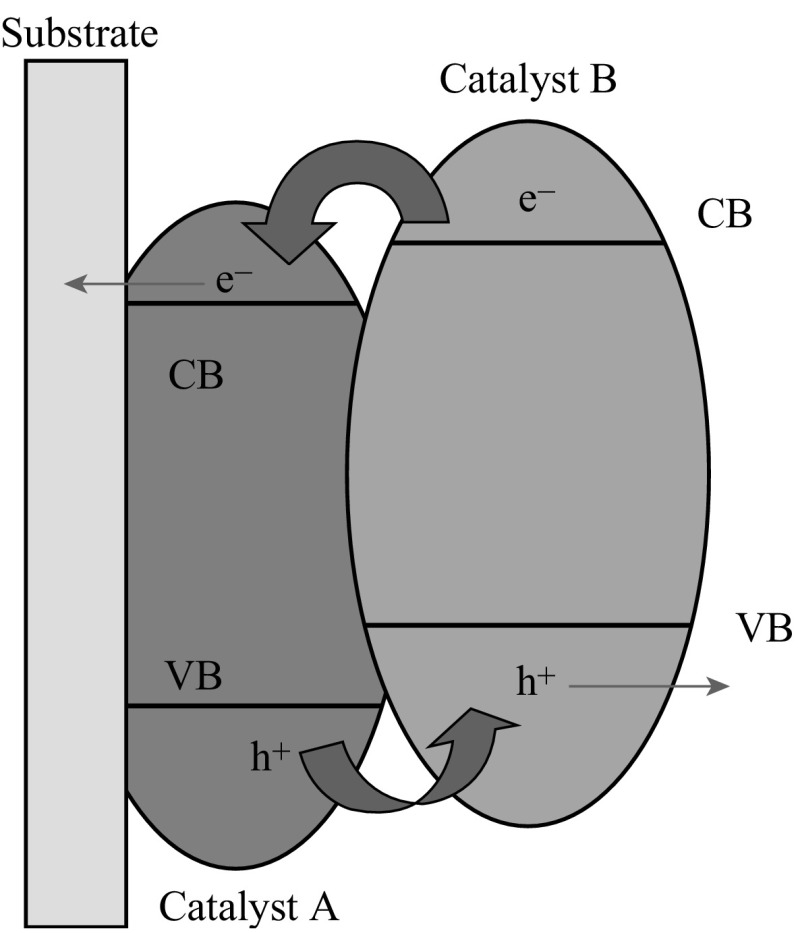
The overlapping in band gaps between two different photocatalysts and the electron-trap mechanism

**Fig. 5:**
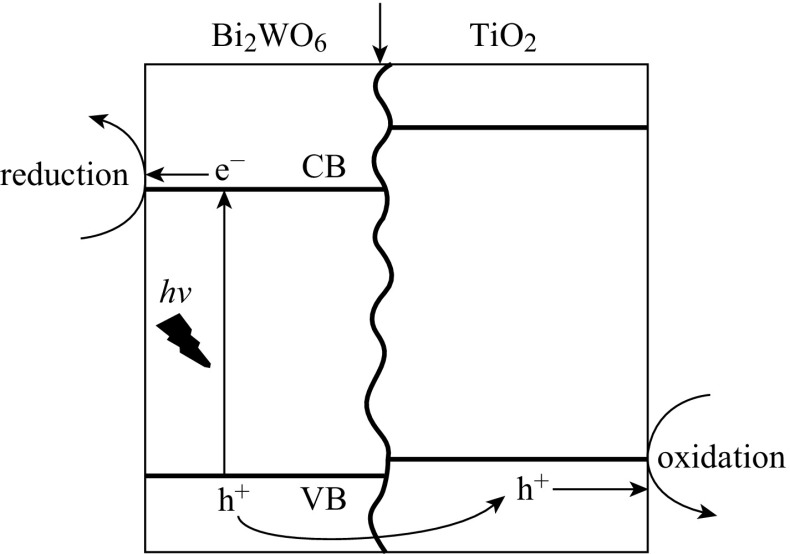
Schematic interfacial electron transfer between TiO_2_ and Bi_2_WO_6_ [47]

Modifying TiO_2_ nano-semiconductor with second nano-semiconductor of lower bandgap to form heterojunction represents another promising route to harvest visible light. The second nano-semiconductor serves as a photosensitizer and builder for internal electric field across the interface. The internal potential bias significantly promotes the excited electrons and holes’ separation and transportation across the interface of the dual photocatalysts, leading to reducing recombination. For instance, TiO_2_ nanotube arrays (TNAs) were always modified with p-type CdTe and Cu_2_O semiconductors [43–45]. TiO_2_ is n-type semiconductor. CdTe and Cu_2_O are p-type semiconductors. Thus, p–n junctions can be formed between them, respectively, which facilitate the separation of the excited electrons and holes. Some heterojunctions can enhance the photoelectrochemical properties because of the overlapping in band gaps between two different photocatalysts, which could favor the charge carrier transfer and separation. Typical examples are the ones illustrated in Figs. 4, 5. The conduction band (CB) of catalyst A (Bi_2_WO_6_) is more negative than the one of catalyst B (TiO_2_); therefore, the excited electrons from catalyst B (TiO_2_) can be quickly transferred to catalyst A (Bi_2_WO_6_). The valance band (VB) of catalyst A (Bi_2_WO_6_) is more positive than the one of catalyst B (TiO_2_), and the excited holes from catalyst A (Bi_2_WO_6_) can be quickly transferred to catalyst B (TiO_2_). As a result, the excited electrons and holes can be separated and transferred quickly for efficient water splitting. This technique has been extensively applied in enhancing the photoelectrochemical performance of TiO_2_ through modification using visible-light responsive semiconductors such as CdS, Bi_2_WO_6_, Rh-doped SrTiO_3_, etc. [46–49].

The above examples are related to binary hybrid systems for PEC water splitting. It should be noted that the CdS–TiO_2_–WO_3_ ternary hybrid system for PEC water splitting has also been reported recently [50]. The cascaded electrons are transferred from CdS to TiO_2_ to WO_3_ through the interfacial potential gradient in the ternary hybrid conduction bands. The maximum photocurrent density of the ternary hybrid is up to 1.6 mA cm^−2^ (at 0 V Ag/AgCl) under visible light irradiation, which is much higher than those of bare CdS and any binary hybrids.

Oxygen vacancy technique has often been applied to enhance the performance of TiO_2_ for PEC water splitting in recent years [51]. Oxygen vacancies can be produced through hydrogen treatment [51, 52]. It can also be generated by annealing metal oxide under oxygen-deficient conditions [53]. In comparison with the hydrogen treatment method, this approach eliminates the potential influence of hydrogen impurities. The PEC performance of TiO_2_ electrodes can be enhanced by controlling the introduction of oxygen vacancies, which as shallow donors can significantly improve electrical conductivities of TiO_2_ nano-semiconductors.

**Fig. 6:**
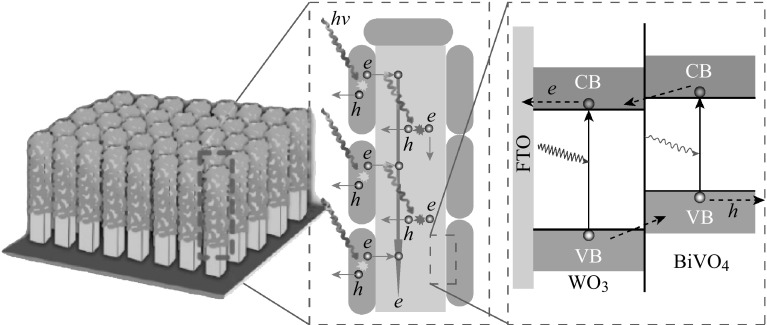
The diagram of BiVO_4_/WO_3_ heterojunction and electron transport process [58]

**Fig. 7:**
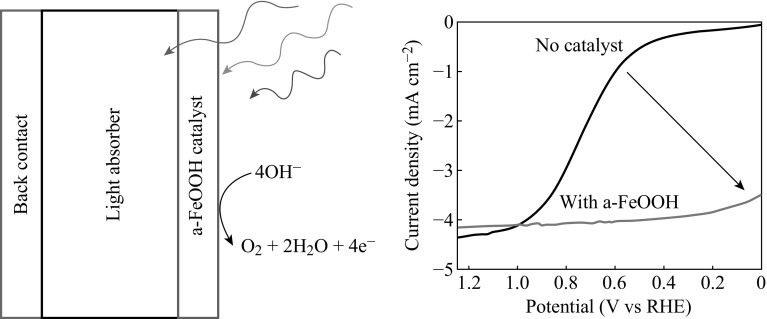
FeOOH as photoanode for photoelectrochemical water splitting [60]

Besides TiO_2_, many other metal-oxide photocatalysts such as BiVO_4_, WO_3_, ZnO, etc. have invariably been attempted to be coated on substrate as photoanodes for PEC water splitting [17, 54–57]. External voltage is needed when BiVO_4_ or/and WO_3_ photoanodes are applied for PEC water splitting. This is because their conduct bands are more positive than the potential of hydrogen evolution. Heterojunction can be applied to improve their photoelectrochemical performance. For example, nanostructured WO_3_/BiVO_4_ heterojunction is prepared for PEC water splitting as shown in Fig. 6 [58]. BiVO_4_ layer was coated on WO_3_ nanorod array by spin coating. The charges can be quickly separated and transferred due to the energy diagram of WO_3_/BiVO_4_ heterojunction. Because of excellent properties of graphene oxides for electron transfer, they have often been used to improve the photoelectrochemical properties of semiconductors in recent years [59]. And some metal hydroxides such as FeOOH (Fig. 7), NiOOH, and layered double hydroxide (LDH) have been reported as effective photoanode materials for PEC water splitting because the alignment reduced interface recombination at the junction between them and semiconductors, and created a more favorable Helmholtz layer potential drop at the semiconductor/electrolyte junction [16, 60, 61], which provides a hint for searching new materials in the field of PEC water splitting.

**Fig. 8:**
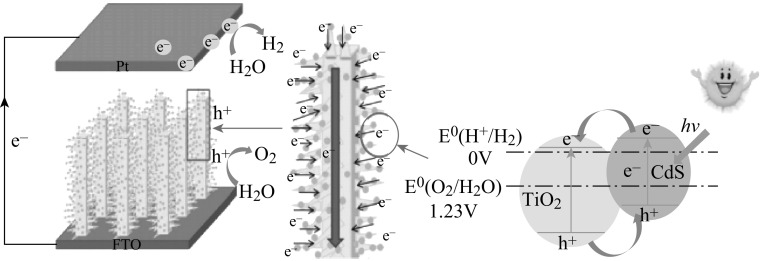
The scheme of the nanostructure of the CdS/TiO_2_ nanoarrays and charge-transfer mechanism [68]

Owing to their narrow bandgap, some n-type metal sulfide [62–64] and oxynitride [65–67] photocatalysts can respond to visible light with long wavelength and appropriate band levels for water splitting, and therefore are chosen as photoanode materials for PEC water splitting in order to make better use of solar energy. CdS (Fig. 8) and other sulfides are often used in photoanodes as sensitizer for PEC water splitting [68]. However, the sulfides are not stable because of photocorrosion, which can be reduced by adding sacrificial agents [69]. Tantalum oxynitrides also show visible light absorption with long wavelength—yet they are not stable and the intensity of absorption is low. Therefore, different techniques such as modification with cocatalysts, heterojunction, etc., have been developed to reduce the photocorrosion and enhance the PEC properties [66–68].

### Photocathode and Cathodic Semiconductors

**Fig. 9:**
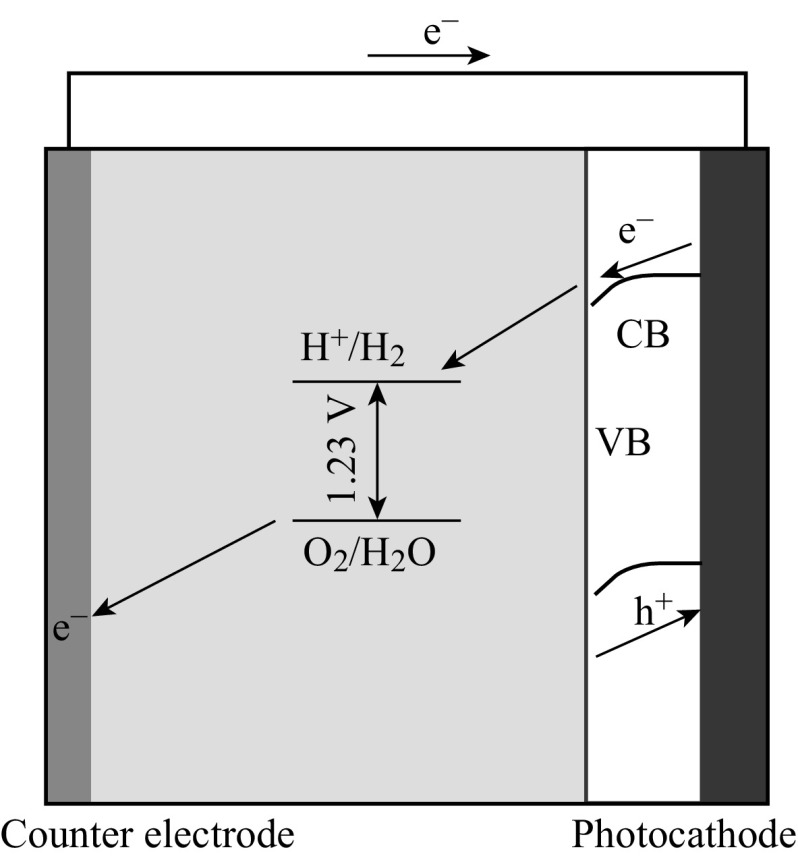
Semiconductors coated on substrates as photocathode for PEC water splitting [15]

Photocathode usually comprises p-type semiconductor coated on conductive substrates in the PEC water splitting system. As shown in Fig. 9, under light illumination, water is reduced on the surface of semiconductor, while water is oxidized on the counter photoelectrode. From the electrochemical potential point of view, the conduction band edge of the photocatalysts should be more negative than the hydrogen evolution potential enabling the photocathode to generate hydrogen. Compared with the reports of n-type semiconductor photoanodes, there are fewer reports based on p-type semiconductor as photocathodes for PEC water splitting [70–77].

**Fig. 10:**
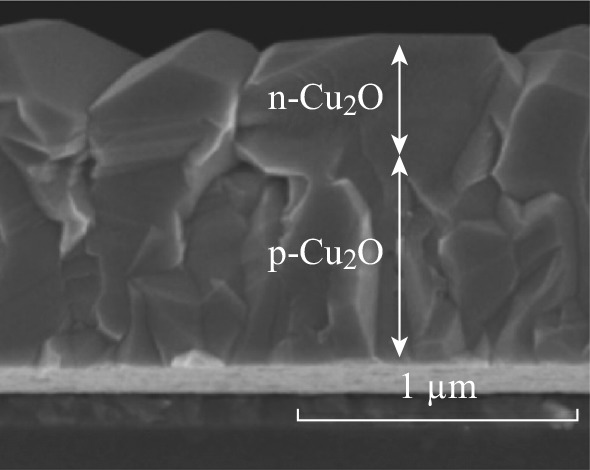
SEM image of a p–n Cu_2_O homojunction [78]

**Fig. 11:**
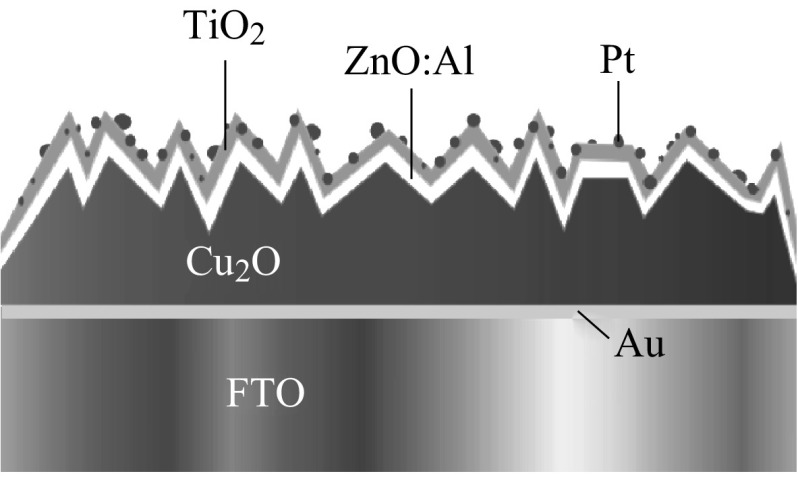
Schematic representation of the electrode structure of the surface-protected Cu_2_O electrode [80]

Cu_2_O is a typical p-type semiconductor used as photoelectrode for PEC water splitting, while it readily gets degraded due to self-reduction by photogenerated electrons. WO_3_/Cu_2_O p–n junctions have been synthesized to reduce the self-reduction of Cu_2_O and enhance the PEC properties [77]. In addition, p–n homojunctions have also been prepared to improve their PEC performance [78, 79]. Colleen et al. [78] fabricated p–n Cu_2_O homojunction solar cells by electrochemically depositing an n-Cu_2_O layer on a p-Cu_2_O layer as shown as in Fig. 10. The intrinsic doping levels of the prepared p-Cu_2_O and n-Cu_2_O layers were very low, and they made Ohmic junctions with Cu metal. The best cell performance (an ŋ of 1.06 %, a *V*_OC_ of 0.621 V, an *I*_SC_ of 4.07 mA cm^−2^, and a fill factor of 42 %) was obtained, which was better than other p–n Cu_2_O homojunctions. Paracchino et al. reported that Cu_2_O photocathode, as shown in Fig. 11, which was protected against photocathodic decomposition in water by nanolayer of Al-doped zinc oxide and titanium dioxide and activated for hydrogen evolution with electrodeposited Pt nanoparticulates, showed photocurrents of up to −7.6 mA cm^−2^ at a potential of 0 V versus the reversible hydrogen electrode at mild pH [80].

B-doped Si with the decoration of Pt, has often been used as a p-type photocathode for PEC water splitting [81, 82], and Pt-modified Si photovoltaic cell (Pt/SiPVC) based on p–n radial junctions with a p-type Si substrate has also been reported as an effective photocathode for PEC water splitting [19]. Si-based photoelectrodes for PEC water splitting has a potential prospect because of low-cost and abundant Si resource, while the efficiency should be further improved.

**Fig. 12:**
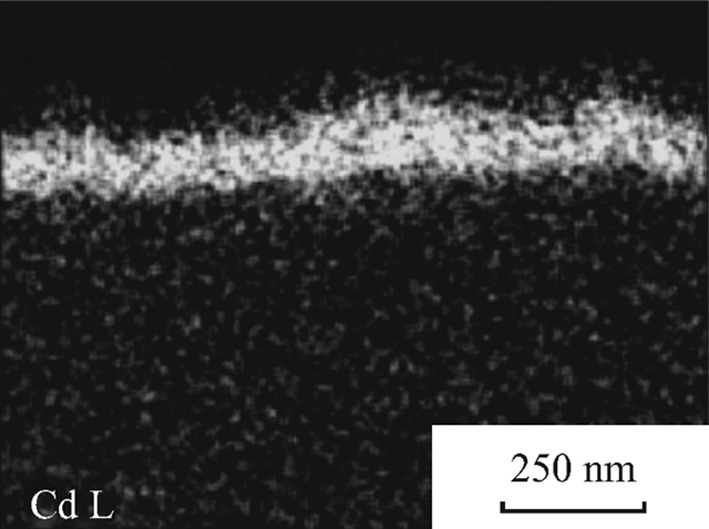
EDX mapping of CdS/CuGaSe_2_ sample with chemical bath deposition for 1 min [18]

Recently, copper sulfides or selenides (CuInS_2_, Cu_2_ZnSnS_4_, Cu(In, Ga)Se_2_, etc.) have been reported as efficient p-type photocathodes for PEC water splitting under visible light [74, 75]. However, they suffered from serious photocorrosion. Moriy et al. deposited CdS on Cu(In, Ga)Se_2_ through chemical bath deposition (CBD) as shown in Fig. 12 [18]. The photocurrent increased due to the p–n junctions formed between them, which accelerated the charge separation. Further, the Pt/CdS/CuGaSe_2_ electrode showed a stable photocurrent (about 4 mA cm^−2^, 0.05 M Na_2_HPO_4_(aq) + 0.05 M NaH_2_PO_4_(aq), pH 7, 150 W Xe lamp, 0 Vvs RHE) under reductive conditions for more than 10 days under visible-light irradiation.

### Z-scheme System for PEC Water Splitting

**Fig. 13:**
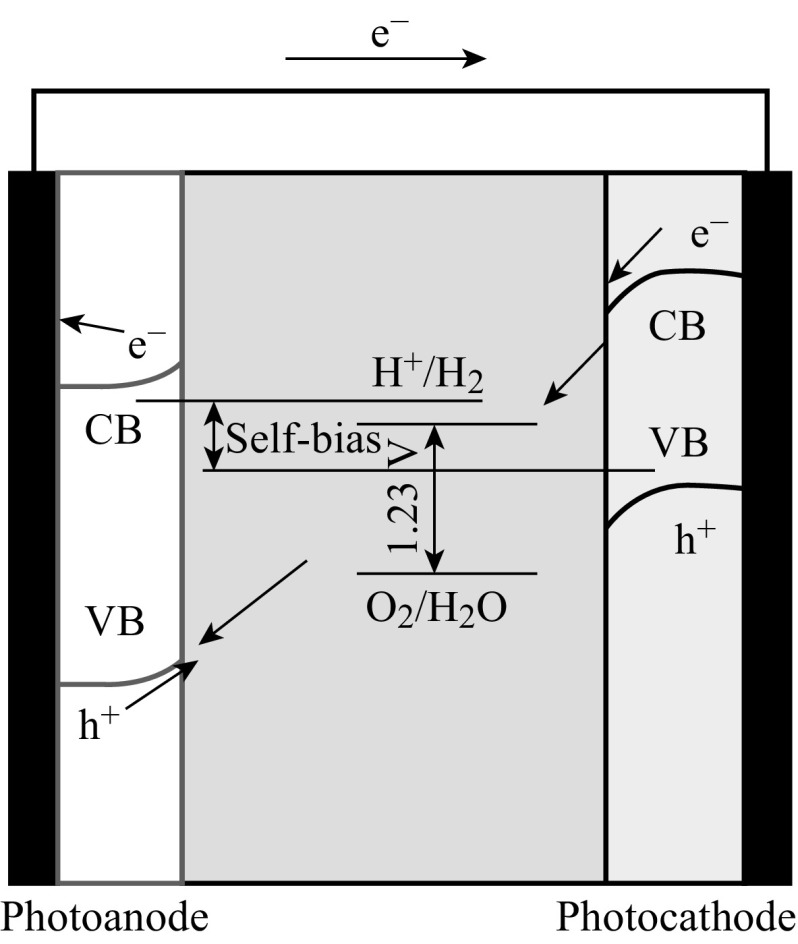
n-type and p-type semiconductors coated on substrates as photoanode and photocathode, respectively, for PEC water splitting (Z-scheme) [15]

With suitable band structure, n-type semiconductors are usually used as photoanode and p-type semiconductors as photocathode for PEC water splitting. It is known that n-type semiconductor has a Fermi level near the conductor band (CB) edge and p-type semiconductor has a Fermi level near the valance band (VB) edge [19]. When n-type and p-type semiconductors are simultaneously used as photoanode and photocathode, respectively, (Z-scheme) as shown in Fig. 13, the mismatching Fermi levels could produce self-bias which can drive the excited electrons from photoanode to combine with the excited holes from photocathode. Meanwhile, water oxidation and reduction take place over the photoanode and photocathode, respectively. In the Z-scheme systems, the self-bias would act as an extra driving force for carriers’ charge transfers and transportation while their performance is still governed by the materials and the competition between chemical reaction and recombination.

**Fig. 14:**
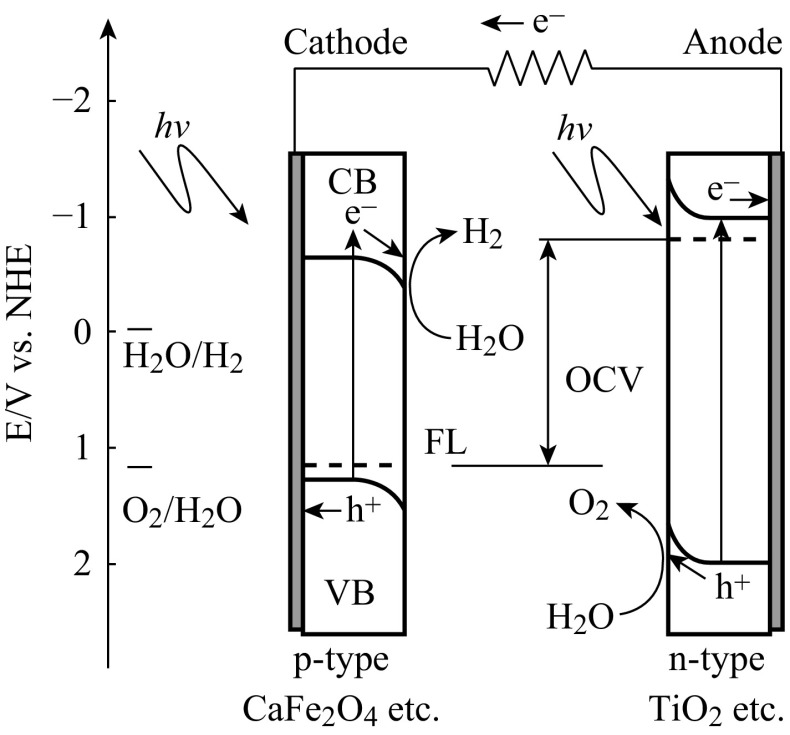
Reaction and band model in photovoltaic cell using p-type CaFe_2_O_4_ and n-type TiO_2_ semiconductor electrodes [83]

**Fig. 15:**
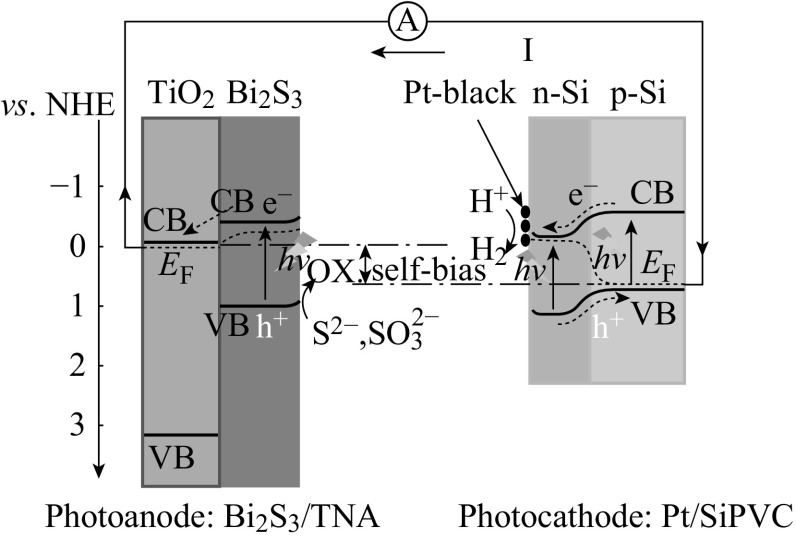
The energy-level diagram of the self-biasing PEC cell assembled with Bi_2_S_3_/TNA photoanode and Pt/SiPVC photocathode under short-circuit situation [19]

Ida et al. constructed a PEC water splitting using p-type CaFe_2_O_4_ as photocathode and n-type TiO_2_ as photoanode [83]. As shown in Fig. 14, under illumination without external voltage, hydrogen and oxygen were produced from photocathode and photoanode, respectively, with a short-circuit current of about 200 μA cm^−2^. However, this system is not responsive to visible light because TiO_2_ only responds to ultraviolet light. A PEC cell [84] made up of WO_3_ photoanode and p-GaInP_2_ photocathode has been reported, both of which are responsive to visible light. However, the conduct band edge of WO_3_ (about 0.25 V vs. NHE) is more positive than the valance band of p-GaInP_2_ (about 0 V vs. NHE), which restricts the charge transfer from photoanode to photocathode via external circuit. Furthermore, the WO_3_ films show relatively poor charge separation properties and weak visible-light absorbance. Thus, this PEC cell cannot split water till enough light intensity is applied. TiO_2_ itself could not respond to visible light. Zeng et al. used Bi_2_S_3_-decorated TiO_2_ nanotube arrays as photoanode and Pt-modified Si photovoltaic cell (Pt/SiPVC) as photocathode [19]. Both of them could respond to visible light. As shown in Fig. 15, the conduction band edge of TiO_2_ (about −0.05 V vs. NHE) is more negative than the valance band edge of Pt/SiPVC (about 0.8 V vs. NHE). A self-bias of about 0.85 V is formed between two photoelectrodes for efficient spontaneous hydrogen evolution and electricity generation under visible light irradiation. However, this system for PEC water splitting needs to be added with sacrificial agents. Thus, an efficient Z-scheme system consisting visible-light responsive photocatalysts for PEC water splitting without addition of sacrificial agents needs to be developed.

## Conclusions and Future Perspective

Photoelectrochemical devices comprising of visible-light-responsive semiconductors have attracted great efforts in water splitting processes to obtain sustainable hydrogen, in search of a promising technique to combat the challenges from global climate change. The prospect of the PEC water splitting systems were highly limited by the nanostructured photocatalysts and their device configurations. The most important issues associated with the PEC devices lay on the sunlight absorbance, energy loss due to undesirable charge carrier recombination and photodegradation of the photoelectrodes. Along with the rapid and great development of nanomaterial science and semiconductor engineering, significant advances have been observed, which shed light on resolving the aforementioned challenges in the PEC water splitting in the recent decades, since the discovery of PEC water splitting by Honda and Fujisma in the 1970s.

Starting from brief introduction of the working mechanism and history of PEC system, this article overviewed the greatest progress in PEC water splitting systems comprising visible-light-responsive photocatalysts. The advantages and disadvantages of the emerging photoanodes and photocathodes were assessed with typical examples, to disclose the potentially effective strategies to promote the efficiency and stability of various PEC systems. Special attention was paid to the TiO_2_ nanotube arrays and modified TiO_2_ photoelectrodes, which can be responsive to visible light via doping with metal and/or nonmetal elements, creating lattice vacancies, and designing heterojunction or Schottky junctions by means of combining other semiconductors or nanoscaled metal particles. Such modifications would not only revise the band structures and energy alignment of TiO_2_ photoelectrodes in the PEC electrolyte solution but also introduce additional drivers that influence photogenerated charge carrier’s separation and transfer. On the basis of advances in TiO_2_ photoelectrodes and PEC systems, other visible-light-responsive semiconductor photoelectrodes and Z-scheme systems were addressed. Among those strategies, heterojunctions and homojunctions were found to be more attractive for single photoelectrode due to the low cost and broad room to align the energy gradient across the semiconductor interface.

The recent advances in the photoelectrodes and their configurations have shed light on resolving the great challenges in PEC water splitting systems, in which one of the very promising solutions could be constructing Z-scheme PEC system involving suitable semiconductor photoanode and photocathode. With reasonable design, the photoexcited charge carriers may be quickly separated and transferred as a result of driven force generated from the self-bias among the separated photoelectrodes. More importantly, semiconductor with small bandgap even responsive to infrared light (heat) could be combined with the Z-scheme systems to harvest full spectrum sunlight. However, the Z-scheme PEC system is still in its early stage and demand further research input due to only limited report on this thriving configuration. The major obstacles in the Z-scheme systems are, similar to the normal PEC system, the efficient (deficient) light harvesting and (in)stability (due to photodegradation) of the semiconductors employed in the photoelectrodes.

Resistance photocorrosion is pivotal to realizing long-term application of PEC water splitting. Although introduction of sacrificial agents may somehow resist photocorrosion of the photoelectrodes, configurations of semiconductor heterojunctions have proven to be more promising because of their broader manufacturing possibility and low cost. In terms of the heterojunction principle, the Z-scheme systems may be stable due to the closed electric circuit involved; however, limited information can be found from the literature regarding their stability, suggesting greater efforts are still required in this area. Apparently, there is a significant step to apply PEC water splitting in hydrogen generation on industrial scale in the near future. However, spurred by global energy and environmental challenge, PEC water splitting is an ideal route to generate hydrogen with less-adverse impact on climate change, and hence, industrial-scale application of PEC water splitting would be the next-door event once the highly efficient and stable photoelectrodes with visible-light-response could be developed, where the most promising PEC system might emerge from the breakthrough on Z-scheme system.
